# Novel Strongly Basic Molecularly Imprinted Solid‐Phase Extraction Sorbent for Simultaneous Determination of Catecholamines and Their Metabolites in Urine

**DOI:** 10.1002/jssc.70467

**Published:** 2026-06-14

**Authors:** Artūrs Šilaks, Antons Podjava, Laura Bernāte, Vladlens Grebnevs, Artur Maciej

**Affiliations:** ^1^ Faculty of Medicine and Life Sciences University of Latvia Riga Latvia; ^2^ Faculty of Chemistry Silesian University of Technology Gliwice Poland

**Keywords:** catecholamines, molecularly‐imprinted polymers, non‐covalent imprinting, strong anion exchange, solid‐phase extraction

## Abstract

Catecholamines are important hormones and neuromediators in the human body. Simultaneous determination of both catecholamines and catecholamine metabolites in bodily fluids can help accurately diagnose dangerous health conditions like adrenal tumors. However, the biggest obstacle is the selective separation of these compounds from the biological matrix. In this work, we propose a novel, dual‐recognition, non‐covalent molecularly‐imprinted polymer that utilizes strong anion exchange as the source of sorbent‐analyte interaction. (4‐Vinylbenzyl)trimethylammonium‐homovanillyl alcohol anion salt and (4‐vinylbenzyl)trimethylammonium‐homoveratric acid anion salt served as template/functional monomer complexes for catecholamines and acidic metabolites, respectively. The sorbent was synthesized using precipitation polymerization and studied with batch adsorption experiments, scanning electron microscopy, Fourier‐transform infrared spectroscopy, and Brunauer–Emmett–Teller surface area and pore size analysis. The polymer was loaded into cartridges and tested with acidified urine samples. The analytes are deprotonated and adsorbed via sorbent's tetraalkylammonium moiety in hydroxide form, which also neutralizes excess acid, removing the need for pH readjustment (which is often necessary for urine analysis). The imprinted sorbent can also be reused at least four times without performance deterioration. Norepinephrine, epinephrine, dopamine, normetanephrine, metanephrine, vanillylmandelic acid, and homovanillic acid were separated and analyzed in a single run using molecularly imprinted solid‐phase extraction combined with liquid chromatography‐tandem mass spectrometry (MISPE‐LC‐MS/MS), with recoveries ranging from 69% (epinephrine) to 97% (homovanillic acid). Method's limits of detection, limits of quantitation, linearity, repeatability, trueness, and intermediate precision were evaluated. Limits of quantitation ranged from 0.7 to 6.5 µg/L (for catecholamines and metanephrines) and from 0.12 to 0.2 mg/L (for acidic metabolites). Compared to commercially available weak‐cation exchange and hydrophilic‐lipophilic balance cartridges, the imprinted sorbent produced stronger catecholamine signals with minimal volume of urine (25 µL). This study successfully demonstrated molecularly imprinted solid‐phase extraction workflow for simultaneous separation and quantitation of urinary catecholamines and their basic and acidic metabolites, proving its compatibility with bioanalysis.

## Graphical abstract

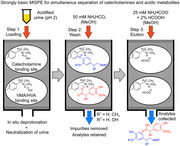

## 1 Introduction

**FIGURE 1:**
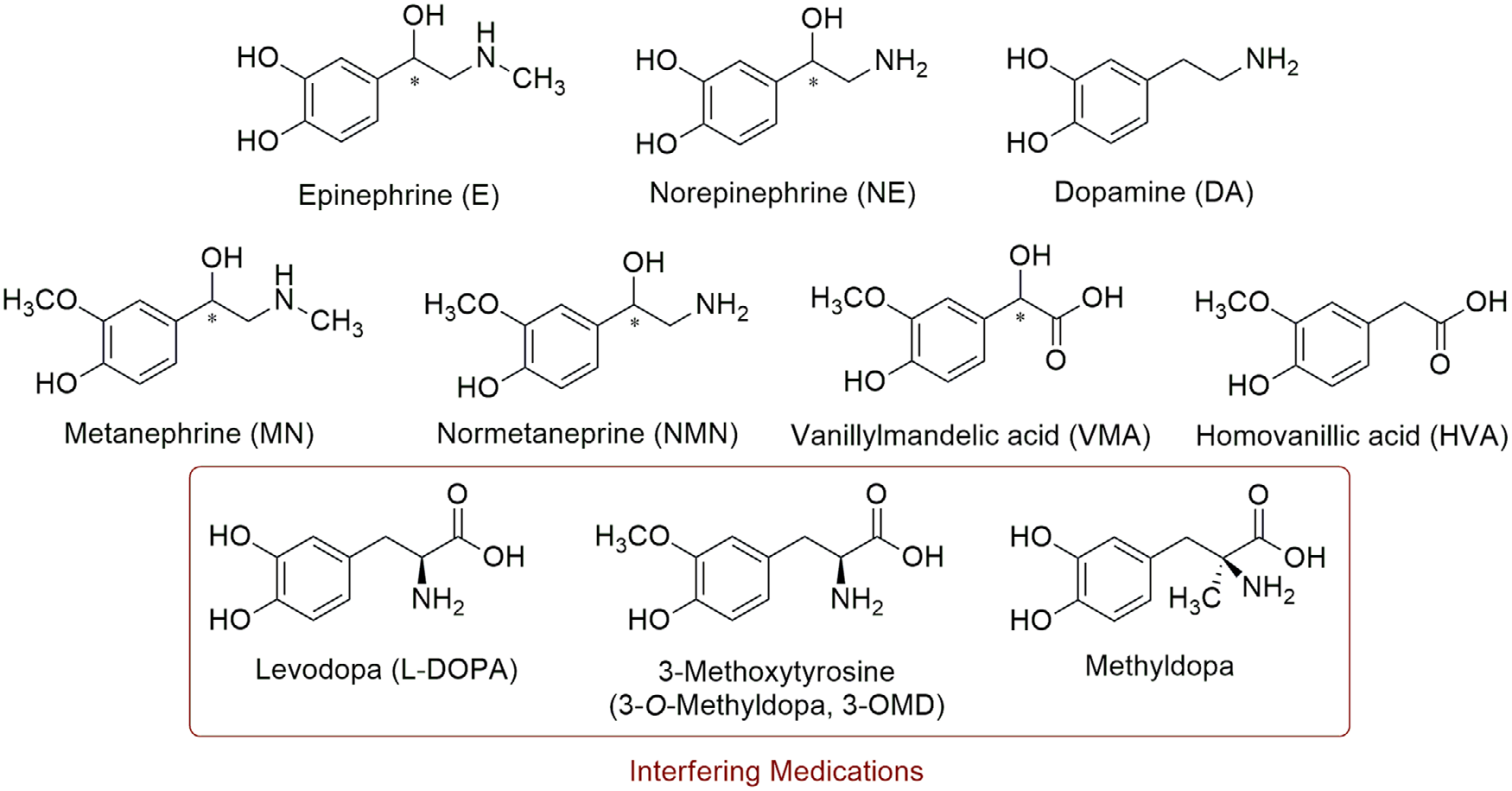
Catecholamines, some of their metabolites, and structurally similar medications of interest.

Catecholamines (CAs), notably norepinephrine (NE), epinephrine (E), and dopamine (DA), are important hormones and neurotransmitters. CAs and catecholamine metabolites (CAMs) are distributed throughout the human body and can be detected in bodily fluids [1]. CAs are metabolized into several intermediary products, including normetanephrine (NMN), metanephrine (MN), dihydroxyphenylglycol (DHPG), and so on. These are further metabolized into vanillylmandelic acid (VMA), homovanillic acid (HVA), and methoxyhydroxyphenylglycol (MHPG), which are excreted in urine [1, 2]. These compounds can provide valuable information on long‐term metabolism and chronic conditions [3]. Abnormal levels of CAs and CAMs are linked to serious health issues, such as cardiovascular diseases, neurodegeneration (Parkinson's and Alzheimer's diseases) [2], as well as benign and malignant tumors such as pheochromocytoma (PHEO), neuroblastoma (NBL), and paraganglioma (PGL) [1, 4]. Simultaneous testing of both CAs and CAMs provides thorough and more reliable medical information. For instance, diagnostic accuracy for NBL reaches 95% if eight key CA/CAM biomarkers are measured simultaneously (NE, E, DA, NMN, MN, 3‐methoxytyramine [3‐MT], VMA, and HVA) [5]. For PHEO, determination of metanephrines (MN and NMN) provides the best results, with Rezkallah et al. reporting 80%–90% sensitivity and 95%–100% specificity for 24‐h urinary metanephrines [6]. Moreover, urinary NE and NMN levels remain largely unaffected in patients taking levodopa (L‐DOPA), a dopaminergic medication [7]. L‐DOPA and methyldopa are important drugs used to combat Parkinson's disease and hypertension, respectively. Administered L‐DOPA is often metabolized into 3‐*O*‐methyldopa (3‐OMD) [8, 9]. These compounds are structurally similar to CAs and have overlapping retention times and precursor‐to‐product ion transitions, which can cause interference and overestimated results. Parent mass commonly used for NE (152 *m*/*z*) is the product ion mass of L‐DOPA (198 *m*/*z* parent ion). A similar overlap exists between methyldopa and E, making analysis difficult for patients taking these medications [10]. 3‐MT is similarly affected by 3‐OMD's interference [9]. Figure 1 shows the structural formulas of CAs, some of their metabolites, and structurally similar interferents.

Modern methods for quantitation of urinary CAs and CAMs include GCMS [1], HPLC with fluorescent and electrochemical detection (ECD) [11], as well as LC‐MS/MS. The latter is the preferred method thanks to its excellent sensitivity, specificity, and the ability to detect many analytes in a single run [12]. But even with these powerful methods, urine samples still require thorough preparation and cleanup due to matrix complexity, very low concentrations of CAs, their chemical instability, and potential interference from structurally similar drugs. Solid‐phase extraction (SPE) is often used for sample treatment in place of more traditional methods (such as liquid–liquid extraction) due to low solvent consumption, higher analyte recovery, and a very wide selection of stationary phases [1, 3]. However, conventional SPE sorbents often have limited selectivity. To solve this, specially designed sorbent materials known as molecularly‐imprinted polymers (MIPs) are being studied, which possess cavities that selectively bind desired analytes using covalent and/or various non‐covalent interactions (hydrogen bonding, electrostatic interactions, π–π stacking, etc.). These materials are well‐suited for a wide variety of uses: detection and separation of biomolecules, pollution monitoring, sensor applications, catalysis, synthesis, and so on. MIPs require four key components: a template molecule, a functional monomer (source of desired interactions), a cross‐linker, and a porogenic solvent. Combining MIP and SPE creates molecularly imprinted solid‐phase extraction (MISPE), offering selectivity, reusability, and flexibility [13, 14]. For instance, MISPE was successfully used by Bouri et al. to analyze NE, E, DA, NMN, and 3‐MT in human urine (but not the acidic metabolites) [15]. Simultaneous isolation of both basic CAs and their acidic metabolites is a significant challenge due to differences in functional groups and polarities. Existing SPE protocols typically rely on non‐selective mixed‐mode sorbents to achieve simultaneous separation. For example, Deng et al. used a weak cation exchange‐mixed‐mode anion exchange (WCX‐MAX) column to develop an online SPE‐MS/MS procedure for quantitation of eight compounds in human urine (E, NE, DA, MN, NMN, 3‐MT, VMA, and HVA) [16]. More recently, Li et al. developed and validated SPE‐LC‐MS/MS with functionalized polymer‐coated silica magnetic beads to determine the same eight compounds in human urine [17]. To our knowledge, there are no recent publications that specifically utilize MISPE to separate CAs, metanephrines, and acidic CAMs simultaneously. The first double‐recognition MISPE protocol by our team was published in 2021 for selective simultaneous isolation of CAs and their metabolites from human plasma with a combination of semi‐covalent and non‐covalent imprinting. While the MIP outperformed non‐imprinted control polymer (NIP) [18], small particle size caused tight packing and considerable column resistance [19], which limited the sorbent to dispersive MISPE. The MIP synthesis and hydrolysis were complicated and time‐consuming. Moreover, some analytes showed only moderate recoveries (up to 58% for NMN and DA). As a result, novel MIP synthesis strategy was developed utilizing non‐covalent imprinting approach to create molecular recognition with the help of electrostatic and in situ generated anion‐exchange interactions between the polymer functional groups and the analytes (CAs, acidic and basic metabolites). The new sorbent provided good solvent flow rate on SPE cartridges as well as satisfactory and selective recovery for both basic and acidic analytes (NE, E, DA, NMN, MN, VMA and HVA) in the presence of common interfering medications like L‐DOPA and methyldopa. To demonstrate the potential for use in bioanalysis, the sorbent was compared to commercially available sorbents and also tested for reusability. The results of the MISPE‐LC‐MS/MS method development and validation for simultaneous determination of urinary CAs and CAMs are presented herein.

## 2 Materials and Methods

The list of all chemicals and solvents used, as well as information on NMR spectroscopy, can be found in Supporting Information.

### 2.1 Synthesis of Functional Monomer‐Template Complexes

#### 2.1.1 Functional Monomer: (4‐Vinylbenzyl)Trimethylammonium Chloride (VBTMA‐Cl)

The reaction was carried out in accordance with our published procedure [18] using 1.41 mL (1.53 g; 10 mmol) of 4‐vinylbenzyl chloride, which yielded 2.09 g (9.9 mmol; 99%) of white‐to‐yellowish crystalline product. ^1^H‐NMR (300 MHz, CD_3_OD, δ): 3.12 (s, 9H), 4.54 (s, 2H), 5.37 (d, 1H, *J* = 11.0 Hz), 5.92 (d, 1H, *J* = 17.6 Hz), 6.81 (dd, 2H, *J* = 17.6, 11.0 Hz), 7.51–7.64 (m, 4H) ppm, which corresponds to published data [18].

#### 2.1.2 Template for CAs and Metanephrines: Homovanillyl Alcohol

The reaction was adapted from a published procedure by Vikingsson et al. [20] using 250 mg of HVA (1.47 mmol) as the substrate. The crude product was purified by column chromatography (normal phase, 40 µm) using petroleum ether–ethyl acetate (4/1, v/v), yielding 221 mg (1.31 mmol, 89%) of homovanillyl alcohol (HVAlc) as an orange viscous liquid. ^1^H‐NMR (300 MHz, CDCl_3_, δ): 2.80 (t, 2H, *J* = 6.45 Hz), 3.83 (t, 2H, *J* = 6.39 Hz), 3.89 (s, 3H), 5.51 (s, 1H), 6.70–6.75 (m, 2H), 6.87 (dd, 1H, *J* = 8.56, 4.65 Hz) ppm, which corresponds to published data [21].

#### 2.1.3 Monomer‐Template Complex for CAs and Metanephrines: (4‐Vinylbenzyl)Trimethylammonium‐Homovanillyl Alcohol Anion Salt (VBTMA‐HVAlc)

A 155 mg of VBTMA‐Cl (0.733 mmol; 1 equiv.) was dissolved in methanol (3 mL) and loaded into a cartridge packed with Ambersep 900 OH^−^ anion exchange resin (1 mL, wetted). The anion exchange resin was washed with methanol until neutral pH was observed. Then, 124 mg (0.733 mmol; 1 equiv.) of HVAlc was added to VBTMA‐OH. The solution was stirred for 1 h, and then concentrated to yield 270 mg (97%) of brown viscous product. ^1^H‐NMR (300 MHz, CD_3_OD, δ): 2.67 (t, 2H, *J* = 7.30 Hz), 3.07 (s, 9H), 3.66 (t, 2H, *J* = 7.31 Hz), 3.76 (s, 3H), 4.47 (s, 2H), 5.37 (d, 1H, *J* = 10.95 Hz), 5.91 (d, 1H, *J* = 17.91 Hz), 6.49–6.68 (m, 3H), 6.80 (dd, 1H, *J* = 17.63, 10.94 Hz), 7,54 (dd, 4H, *J = *21.63, 8.19 Hz) ppm. ^13^C‐NMR (75 MHz, CD_3_OD, δ): 135.6, 132.8, 130.1, 127.8, 127.4, 126.8, 126.6, 121.0, 115.2, 114.9, 112.3, 63.2, 56.5, 54.4, 51.7, 38.4 ppm. See Supporting Information for IR and HRMS spectra.

#### 2.1.4 Monomer‐Template Complex for Acidic CAMs: (4‐Vinylbenzyl)Trimethylammonium‐Homoveratric Acid Anion Salt (VBTMA‐DMPAA)

A 500 mg of VBTMA‐Cl (2.36 mmol; 1 equiv.) was dissolved in methanol (10 mL) and converted to its OH^−^ form in the same manner as before with 3 mL of anion exchange resin. Then, 463 mg (2.36 mmol; 1 equiv.) of 3,4‐dimethoxyphenylacetic acid (DMPAA, template for acidic CAMs) was added to VBTMA‐OH solution. The solution was stirred for 1 h, and then concentrated to yield 940 mg (107%) of yellow viscous product. ^1^H‐NMR (300 MHz, CD_3_OD, δ): 3.09 (s, 9H), 3.40 (s, 2H), 3.78 (d, 3H), 3.81 (d, 3H), 4.49 (s, 2H), 5.38 (d, 1H, *J = *11.0 Hz), 5.91 (d, 1H, *J = *17.6 Hz), 6.75–6.87 (m, 3H), 6.96 (s, 1H), 7.47–7.64 (m, 4H) ppm. ^13^C‐NMR (75 MHz, CD_3_OD, δ): 179.8, 150.2, 148.9, 141.5, 137.0, 134.2, 132.2, 128.3, 128.0, 122.6, 116.6, 114.4, 113.0, 70.2, 56.5, 53.0, 45.6 ppm. See Supporting Information for IR and HRMS spectra.

### 2.2 Sorbent Synthesis and Activation

#### 2.2.1 Optimized Synthesis of Molecularly‐Imprinted Polymeric Sorbent (MIP)

A 0.446 g (1.2 mmol) of VBTMA‐DMPAA, 0.412 g (1.2 mmol) of VBTMA‐HVAlc, and 3.70 g (24.0 mmol) of *N,N'*‐Methylenebisacrylamide (MBAA, cross‐linker) were dissolved in acetonitrile‐dimethylformamide‐methanol (ACN/DMF/MeOH) mixture (115.8 mL ACN, 29.3 mL of MeOH, and 22.0 mL of DMF), cooled to 0°C, and thoroughly degassed with argon. Then, 0.880 g of AIBN was dissolved separately in 8.80 mL of degassed ACN and then immediately added to the reaction mixture. The reaction flask was sealed and stirred for 5 h at 65°C. The product was separated by centrifugation (10 min at 2880×*g*), washed three times with 80 mL of acidic solvent (50 mM HCl in ACN/water 1/1 (v/v) mixture) and with methanol (3 × 80 mL). All washing supernatants were analyzed by HPLC‐DAD (analysis method described in Supporting Information). If template or monomer residues were observed, the washing was repeated. The product was dried at room temperature. A yellowish powder was obtained in quantitative yields.

#### 2.2.2 Optimized Synthesis of Non‐Imprinted Polymeric Control Sorbent

A 0.0635 g (0.300 mmol) of VBTMA‐Cl and 0.463 g (3.00 mmol) of MBAA were dissolved in ACN/DMF/MeOH mixture (14.5 mL ACN, 3.66 mL of MeOH, and 2.75 mL of DMF). The reaction mixture was cooled to 0°C and degassed with argon. Then, 0.110 mg of AIBN was dissolved separately in 1.10 mL of degassed ACN and then immediately added to the reaction mixture. The reaction flask was sealed and stirred for 5 h at 65°C. The polymer was processed and dried in the same manner as MIP (10 mL scale used for washing). A white powder was obtained in quantitative yield.

#### 2.2.3 Sorbent Conversion to Hydroxide Form (OH^−^Activation)

A 250 mg of dried MIP or NIP was suspended in 12.5 mL of 1 M NaOH solution in MeOH/water (1/1, v/v) in a screw‐capped vial. The suspension was stirred for 24 h at 250 RPM using an orbital shaker. The polymer was separated by centrifugation and washed with MeOH/water (1/1, v/v) (3 × 12.5 mL) and pure methanol (3 × 12.5 mL or until neutral pH was observed). Sorbent suspension was made in methanol (20 mg/mL concentration) for SPE and competitive binding experiments.

### 2.3 MIP Optimization and Selectivity Studies

CA and drug stock solutions for NE, E, DA, NMN, VMA, HVA, salbutamol, dobutamine, ibuprofen (10 mM each), and MN (21 mM) were made by dissolving the starting compounds in methanol. Calibration solutions were made in 1–15 µM range by combining the stock solutions, evaporating methanol, and diluting them with 0.1% formic acid in water (v/v). SPE sample solution for selectivity testing was made by combining the stock solutions, evaporating the solvent, and diluting the mixture to 10 µM with ACN/water (4/1, v/v) or pure water. SPE washing solution was ACN/MeOH (1/1, v/v). SPE eluting solution was made by dissolving formic acid (2%, v/v) and ammonium formate (AmForm) (25 mM) in methanol.

A 24‐position SPE manifold was used for MISPE experiments. A 3 mL SPE cartridge was loaded with 20 mg of activated sorbent. Polypropylene frit with 0.45 µm nylon filter membrane was placed in the cartridge. Sorbent suspension was sonicated for 1 min, loaded into the cartridge, and compacted. Another polypropylene frit was placed on top. The cartridge was conditioned for SPE by methanol (3 mL) and then by 3 mL of ACN/water 4/1 (v/v) or pure water, depending on sample solvent. A 300 µL of sample solution was passed through the cartridge, followed by 500 µL of washing solution and then 2 mL of eluting solution (in 500 µL portions). All fractions were evaporated using a sample concentrator (nitrogen blower), diluted to 500 µL with aqueous 0.1% formic acid solution (v/v) and then analyzed using Shimadzu Prominence HPLC‐PDA (see Supporting Information).

SPE recovery values of CAs and their metabolites were compared to the recovery of sterically hindered reference medications (NE, E and DA compared to dobutamine; MN and NMN to salbutamol; VMA and HVA to ibuprofen, respectively). Performance of each MIP was then compared to its respective NIP using selectivity factor (SF), calculated according to Equation 1:

(1) $$SF\;=\frac{Rec_{CA\left(MIP\right)}/Rec_{Ref\left(MIP\right)}}{Rec_{CA\left(NIP\right)}/Rec_{Ref\left(NIP\right)}}\;$$

where *Rec_CA_* denotes analyte recovery; *Rec_Ref_* is reference substance recovery (dobutamine, salbutamol, or ibuprofen) on MIP or NIP.

### 2.4 Sorbent Physiochemical Characterization

Detailed information about HPLC/PDA system, FT‐IR and NMR spectrometers, scanning electron microscopy (SEM), and BET analysis is provided in Supporting Information.

#### 2.4.1 Static Binding Experiments

DA·HCl and VMA stock solutions (10 mM and 1 mM) were prepared by dissolving the starting compounds in methanol. Calibration solutions in 1–500 µM concentration range were made by evaporating an appropriate volume of stock solution and diluting it to 1 mL with 0.1% formic acid in water (v/v). Samples at all concentration levels were prepared in triplicate. Binding capacity and affinity of DA and VMA were tested for the optimized MIP and its corresponding NIP. Activated sorbent suspension was sonicated for 15 min. Then, 25 µL of suspension (0.5 mg of sorbent) was transferred to 1.5 mL Eppendorf tubes. Appropriate volume of DA or VMA solution was added. The mixture was diluted to 250 µL with methanol. Then, 750 µL of ACN was added. The sample was placed into orbital shaker for 24 h (200 RPM), then centrifuged. A 500 µL of supernatant was transferred into a vial, evaporated, diluted with 0.1% formic acid solution in water (v/v), and analyzed using Agilent 1290 Infinity II UPLC‐DAD system (see Supporting Information for details). Equilibrium binding capacities of VMA and DA on MIP or NIP (*Q*, nmol/mg) were calculated using Equation 2:

(2) $$Q=\frac{\left(C_{i}\;-\;C_{e}\right)}{m}\;V$$

where *C_i_* denotes the initial analyte concentration (nmol/mL), *C_e_* is the equilibrium concentration (nmol/mL), *m* is the sorbent aliquot mass (mg) and *V* is the total volume of the solution (mL).

The obtained values of *Q* were plotted against *C_e_* and then fitted to Langmuir (Equation 3) and Freundlich (Equation 4) models [22 p. 87].

(3) $$Q=\frac{Q_{max}\cdot K\cdot C_{e}}{1+K\cdot C_{e}}\;$$

(4) $$Q=\;K\cdot C_{e}^{\alpha }$$

where *Q* is the equilibrium adsorption capacity (nmol/mg), and *Q_max_* is the sorbent's maximum adsorption capacity according to the respective model (nmol/mg). *K* (mL/nmol) denotes the binding equilibrium constant, which measures analyte's affinity per binding site. *C_e_* is the equilibrium concentration (nmol/mL). *α* is the heterogeneity index (0 < *α *< 1), where 0 denotes an increasingly heterogeneous surface and 1 describes a perfectly smooth surface with homogeneous adsorption site energetics [23]. The results were fitted using nonlinear regression in IBM SPSS Statistics 22.

#### 2.4.2 Dynamic Binding Experiments

Preparation of sorbent suspension and DA/VMA stock solutions was identical to static binding experiments. 25 µL of the sorbent suspension was transferred to 1.5 mL Eppendorf tubes. Then, 20 µL of DA·HCl or VMA solution (20 µM in MeOH) was added, followed by 955 µL of ACN/MeOH mixture (4.66/1, v/v) to ensure precise ACN/MeOH 4/1 (v/v) ratio later. The samples were placed into orbital shaker and mixed for 0.5, 1, 2, 3, 4, 5, and 24 h and then centrifuged. Then, 500 µL of supernatant from each sample was poured into a vial, evaporated, diluted with 0.1% aqueous solution of formic acid (v/v), and analyzed with the same HPLC method as static binding.

### 2.5 MISPE‐HPLC‐MS/MS Method Optimization

#### 2.5.1 Solution Preparation

NE, E, DA, NMN, VMA, HVA, 3‐OMD, methyldopa, L‐DOPA (10 mM each), and MN (21 mM) stock solutions were made by dissolving the starting compounds in methanol. Analyte spiking solution was made by combining the stock solutions and diluting them with methanol in 0.37–47.6 mg/L range. Deuterated internal standard solution for CAs was made by combining the factory 100 mg/L solutions of NE‐D6, E‐D6, DA‐D4, NMN‐D3, and MN‐D3 and then diluting them with methanol to achieve 1–3.3 mg/L range for use in spiking. VMA‐D3+HVA‐D5 internal standard solution was prepared separately by combining both factory 100 mg/L solutions in 1/1 (v/v) ratio without dilution.

#### 2.5.2 Urine Sample MISPE Processing

An anonymized pooled urine sample for MISPE was provided by E. Gulbis Laboratory LLC (Riga, Latvia), acidified to pH 2 with HCl, and stored at 4°C in the dark. Pooled urine was centrifuged (1440×*g* for 5 min), then 25 µL urine aliquot was transferred into an Eppendorf tube and spiked with 2.5 µL of analyte solution (if pre‐spiked), 5 µL of deuterated CA standard solution, and 5 µL of VMA‐D3+HVA‐D5 standard solution. Urine was then diluted with 25 µL of water and 200 µL of selected solvent. Spiked SPE sample concentrations were as follows: 5.1 µg/L of NE; 3.7 µg/L of E; 30.6 µg/L of DA; 5.5 µg/L of NMN; 3.9 µg/L of MN; 0.48 mg/L of VMA; 0.44 mg/L of HVA; 0.20 mg/L of L‐DOPA; 0.21 mg/L of methyldopa; and 0.21 mg/L of 3‐OMD. SPE experiments were carried out in 3 mL SPE cartridges with 60 mg load of optimized MIP. The cartridge was conditioned with 3 mL of methanol, followed by 3 mL of the solvent that matches the SPE sample (solvent/water 4/1, v/v). Urine sample was passed through the cartridge, washed with 500 µL of a selected washing solution, and then eluted with 1000 µL of a selected eluting solution. Analyte spiking solution (2.5 µL) was added to post‐spike samples. The fractions were evaporated and diluted to 250 µL with 0.1% (v/v) formic acid solution in water. SPE analyte recovery was calculated by comparing analyte signal intensities of pre‐spiked eluate to post‐spike signal intensities (both signals were normalized against their respective deuterated internal standards).

#### 2.5.3 MISPE Cartridge Regeneration

A 6 mL of 5% HCOOH solution in methanol (v/v) was passed through the cartridge to remove any remaining impurities, followed by 6 mL of pure methanol. The sorbent was recharged with 6 mL of caustic agent (1 M NaOH with 1 M ammonia in MeOH/water 1/1, v/v). Caustic solution was removed with 6 mL of water. Regenerated cartridge was conditioned and used as before.

#### 2.5.4 Processed Urine Sample Analysis

Processed samples were analyzed using an Agilent 1290 Infinity II UHPLC‐MS/MS System (G7104A flexible quaternary pump, G7167B multisampler, G7116B multicolumn thermostat, and Agilent 6470 QQQ mass spectrometer). Optimized ion source conditions were as follows: 300°C gas temperature, 9 L/min gas flow, 40 psi nebulizer pressure, 350°C sheath gas temperature, 8 mL/min sheath gas flow. Data were acquired in MRM mode and analyzed using Agilent MassHunter and Agilent Quantitative Analysis Tool (QuantMyWay). Zorbax Eclipse Plus C18 column (1.8 µm, 3.0 × 50 mm) was used for LC separation. Mobile phases: 0.1% (v/v) formic acid in water (A), and 0.1% (v/v) formic acid in methanol (B). The following gradient was used: 0.0–1.1 min, 0% B (to waste), 0.2 mL/min; 1.1–3.48 min, to 10% B, 0.3 mL/min; 3.48–5.48 min, 32% B, 0.3 mL/min; 5.48–8.48 min, to 100% B, 0.3 mL/min; 8.48–10.48 min, 100% B, 0.3 mL/min (to waste). The most important MS/MS parameters (fragmentor voltage, collision energy, and analyte MRM transitions) were optimized using Agilent's built‐in MS Optimizer and Source Optimizer. Used MRM transitions are listed in Table 1.

**TABLE 1:** Multiple reaction monitoring transitions were used for the detection of studied catecholamines, their metabolites, and interfering medications in human urine.

Analyte	Precursor Ion, *m*/*z*	Product Ion, *m*/*z*	Fragmentor voltage, V	Collision energy, V	Cell accelerator voltage, V	Capillary voltage, V
NE	152.0 (170.1)^a^	107.0 (152.0)^a^	117 (59)^a^	20 (4)^a^	4 (5)^a^	+2500^b^
NE‐D6	176.1 (158.0)	111.0 (111.0)	59 (117)	20 (20)	5 (5)	
E	184.1 (166.0)	166.0 (107.0)	73 (132)	8 (20)	5 (4)	
E‐D6	190.1	172.0	73	8	5	
NMN	166.0 (166.0)	121.0 (134.0)	117 (117)	16 (16)	5 (4)	
NMN‐D3	169.0	137.0	117	16	5	
DA	154.1 (137.0)	137.0 (91.1)	78 (132)	8 (20)	4 (4)	
DA‐D4	158.1	141.0	78	8	5	
MN	198.1 (180.0)	180.0 (148.0)	73 (127)	8 (16)	4 (4)	
MN‐D3	183.0	151.0	127	16	5	
L‐DOPA	198.0	152.0	80	13	4	
Methyldopa and 3‐OMD	212.1	166.0	90	12	5	
VMA	197.0 (197.0)	138.0 (137.0)	117 (117)	8 (24)	4 (5)	−3500
VMA‐D3	200.0	140.0	117	24	5	
HVA	137.0 (181.1)	122.0 (137.0)	115 (73)	8 (0)	2 (4)	
HVA‐D5	142.0	127.0	115	8	5	

^a^ Qualitative transitions and their settings are given in parentheses.

^b^ The “+” or “−” sign denotes ESI ionization mode.

#### 2.5.5 SPE With WCX and Hydrophilic‐Lipophilic Balance

The developed MISPE procedure was compared to commercially available hydrophilic‐lipophilic balance (HLB) and WCX cartridges using non‐spiked pooled urine. HLB SPE was performed according to the published procedure by Shen et al. [24]. Waters Oasis HLB cartridge (3 mL/60 mg) was conditioned with 1 mL of methanol and then with 1 mL of water. A 500 µL aliquot of pooled urine was diluted to 1 mL with water, thoroughly mixed and applied onto the cartridge. The sample was washed with 1 mL of water, eluted with 1 mL of 0.1% (v/v) of aqueous formic acid solution, and then analyzed. WCX was performed according to the proprietary procedure used by E. Gulbis Laboratory LLC: 200 µL of the same non‐spiked urine was applied on a 3 mL WCX cartridge; final processed sample volume was 500 µL. All samples were analyzed using the same LC‐MS/MS procedure. The results (TIC MRM and individual MRM transitions) were compared to the optimized MISPE procedure.

### 2.6 MISPE‐HPLC‐MS/MS Method Validation

Lyophilized urine calibrators and quality controls (QCs) were used for validation. Lyophilized calibrators for CAs/free metanephrines/VMA/HVA/5‐HIAA in urine (Levels 1–6, LOT 010) were obtained from Eureka/Sentinel Diagnostics (Chiaravalle, Italy). Each calibrator level was reconstituted with 1 mL of deionized water, divided into 250 µL portions, and stored in brown glass vials at −20°C in the dark. Analyte concentrations in calibrators are shown in Table S1 (in Supporting Information), as provided by Eureka (5‐HIAA not shown).

Lyophilized urinary QC for CAs, free metanephrines, and serotonin (Level 1 and Level 2, LOT 3023), as well as lyophilized urinary QC for VMA, HVA, and 5‐HIAA (Level 1 and Level 2, LOT 2623), were purchased from Chromsystems Instruments & Chemicals GmbH (Gräfelfing, Germany). Each QC level was reconstituted with deionized water according to the instructions (2 mL for CAs and MNs, 1 mL for acidic metabolites), divided into 250 µL portions, and stored in brown glass vials at −20°C in the dark. Target QC concentrations are shown in Table S2 (in Supporting Information), as provided by Chromsystems (serotonin and 5‐HIAA are not shown).

A 25 µL calibrator or QC aliquot was transferred into an Eppendorf tube. Then, 5 µL of deuterated CA standard solution and 5 µL of VMA‐D3+HVA‐D5 standard solution were added. The calibrator or QC was diluted with 25 µL of water and 200 µL of 5 mM ascorbic acid (AsA) solution in methanol, and then mixed. The sample was passed through the cartridge, washed with 500 µL of 50 mM ammonium bicarbonate (AmBic) solution in methanol, and then eluted with 1000 µL of the acidic solution (25 mM AmForm and 2% (v/v) formic acid in methanol). The fractions were evaporated and diluted to 250 µL with 0.1% formic acid solution in water (v/v). Processed calibrators and QCs were analyzed with Agilent Infinity II UPLC‐MS/MS using the same method as before. Method's linear regression, weighting factor, repeatability, intermediate precision, and trueness were calculated using Agilent Quantitative Analysis software. Method's limit of detection (LOD) and limit of quantitation (LOQ) were determined by diluting Level 1 and Level 0 calibrators until respective signal‐to‐noise (SNR) ratios were reached for each analyte (SNR 3 for LOD; SNR 10 for LOQ).

## 3 Results and Discussion

### 3.1 MIP Design and Optimization

**FIGURE 2:**
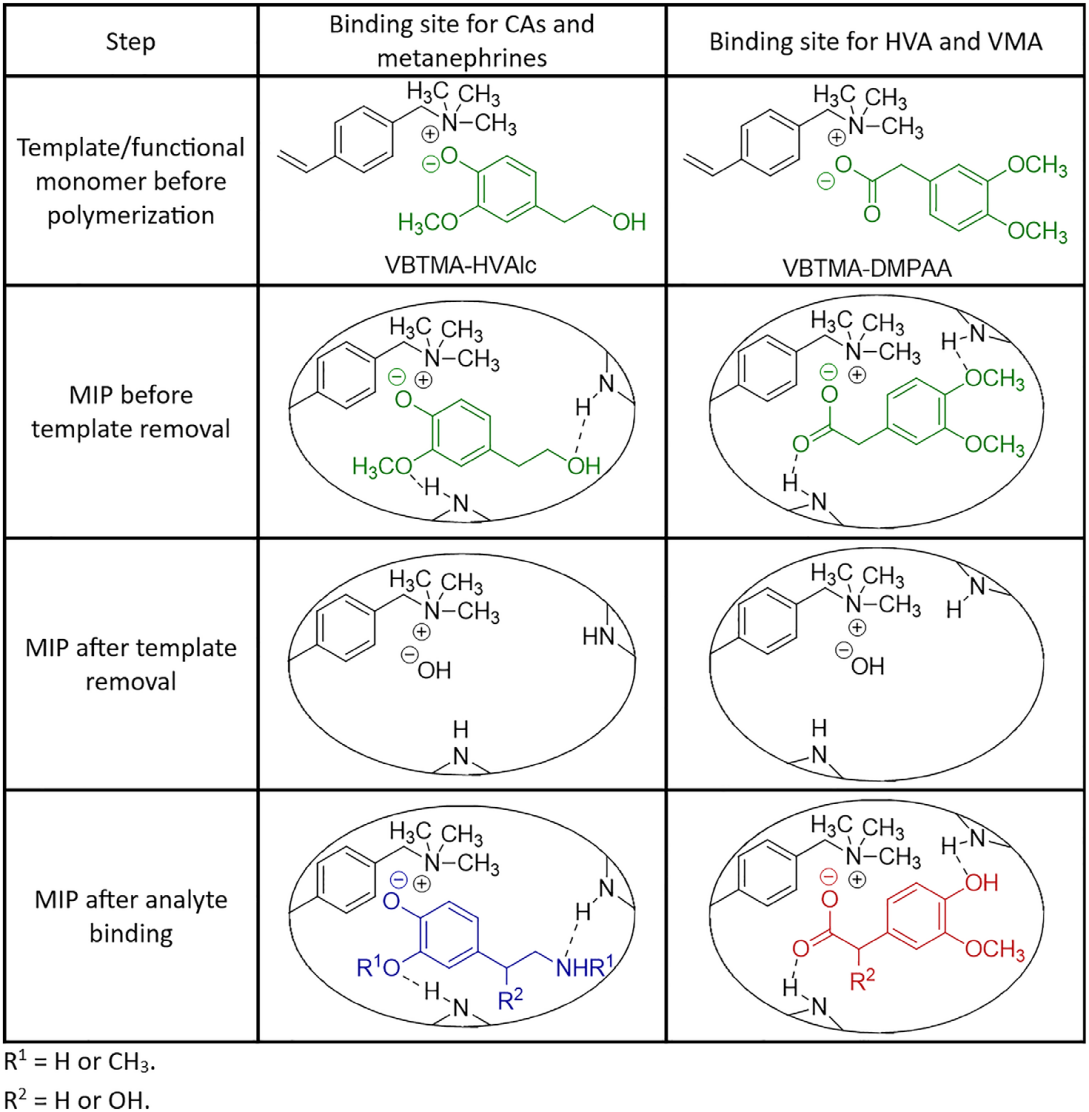
Functional monomers/template complexes and proposed binding sites of the molecularly‐imprinted polymer.

Polymers made using our previous semi‐covalent imprinting strategy caused difficulties, including lengthy synthesis (> 48 h), incomplete hydrolysis of 3‐phenylpropyl acrylate (3‐PPA) functional monomer/template (≤ 50%), and small particle size, resulting in low flow rates and reduced analyte recoveries [18, 19]. In response, a new polymer synthesis strategy was devised that relies on non‐covalent interactions, namely electrostatic attraction and hydrogen bonding, which together have a synergistic effect on analyte binding [25]. HVAlc and DMPAA were chosen as templates for CAs and acidic CAMs, respectively, due to their chemical stability and structural similarity to the analytes. (4‐Vinylbenzyl)trimethylammonium (VBTMA) moiety is the source of electrostatic interaction and is used to create template/functional monomer complexes for both analyte groups. VBTMA‐DMPAA forms through simple electrostatic interaction between DMPAA's carboxylic group and VBTMA's tetraalkylammonium moiety, while VBTMA‐HVAlc is created by deprotonating the template's free phenolic group. Both complexes are easy to make, do not require hydrolysis, and are stable under polymerization conditions. As all analytes have at least one free phenolic OH group that is weakly acidic (pK_a_ 8–10) [26, 27], our sorption strategy is based on the fact that replacing both templates in the sorbent cavities by hydroxide ions leads to formation of a strongly basic anion exchanger capable of in‐situ deprotonation of the analytes and their subsequent retention, with additional interactions provided by hydrogen bonding between the sorbent and the analyte side chain. Figure 2 shows intended binding sites and sources of interactions in the new sorbent.

The potential for practical use of anion exchange with phenolic compounds was investigated by Grant et al., who separated morphine from non‐phenolic codeine by deprotonating and adsorbing the former on a quaternary ammonium anion exchanger [28]. Chu et al. later isolated halogenated phenolic compounds from bovine plasma and serum using Oasis MAX SPE [29]. Our own preliminary tests were carried out with Ambersep 900 OH^−^ strong anion exchange resin: a solution of CAs and MNs (10 µM each in ACN/water 4/1 v/v and pure water) was loaded onto the Ambersep and then quickly eluted. Medium to high analyte recovery was observed, thus proving the possibility of using strong anion exchange to adsorb CAs.

To implement our new imprinting strategy, the sorbent had to satisfy three key parameters: high sorption selectivity, adequate analyte recovery, and good permeability to solvents under cartridge SPE conditions. MIP optimization screening was carried out with different crosslinker‐to‐monomer molar ratios (20:1 to 2:1), solvents (ACN, MeOH, and DMF), and two radical initiators with variable loadings (3%–24% of AIBN or V‐65, by dry weight). Corresponding NIPs were synthesized as well (both templates replaced by an equivalent amount of VBTMA‐Cl). Each MIP/NIP pair was tested under SPE conditions using standard analyte mixture prepared in either ACN/water 4/1 (v/v) or pure water to determine analyte recovery. ACN/water 4/1 (v/v) was chosen as a medium polar system due to its use in bioanalysis as well as its resemblance to the solvent used during synthesis (analyte binding usually benefits from similar solvation that was used for polymerization) [30], while water acted as a polar solvent with strong influence of hydrogen bonding and electrostatic interactions.

**FIGURE 3:**
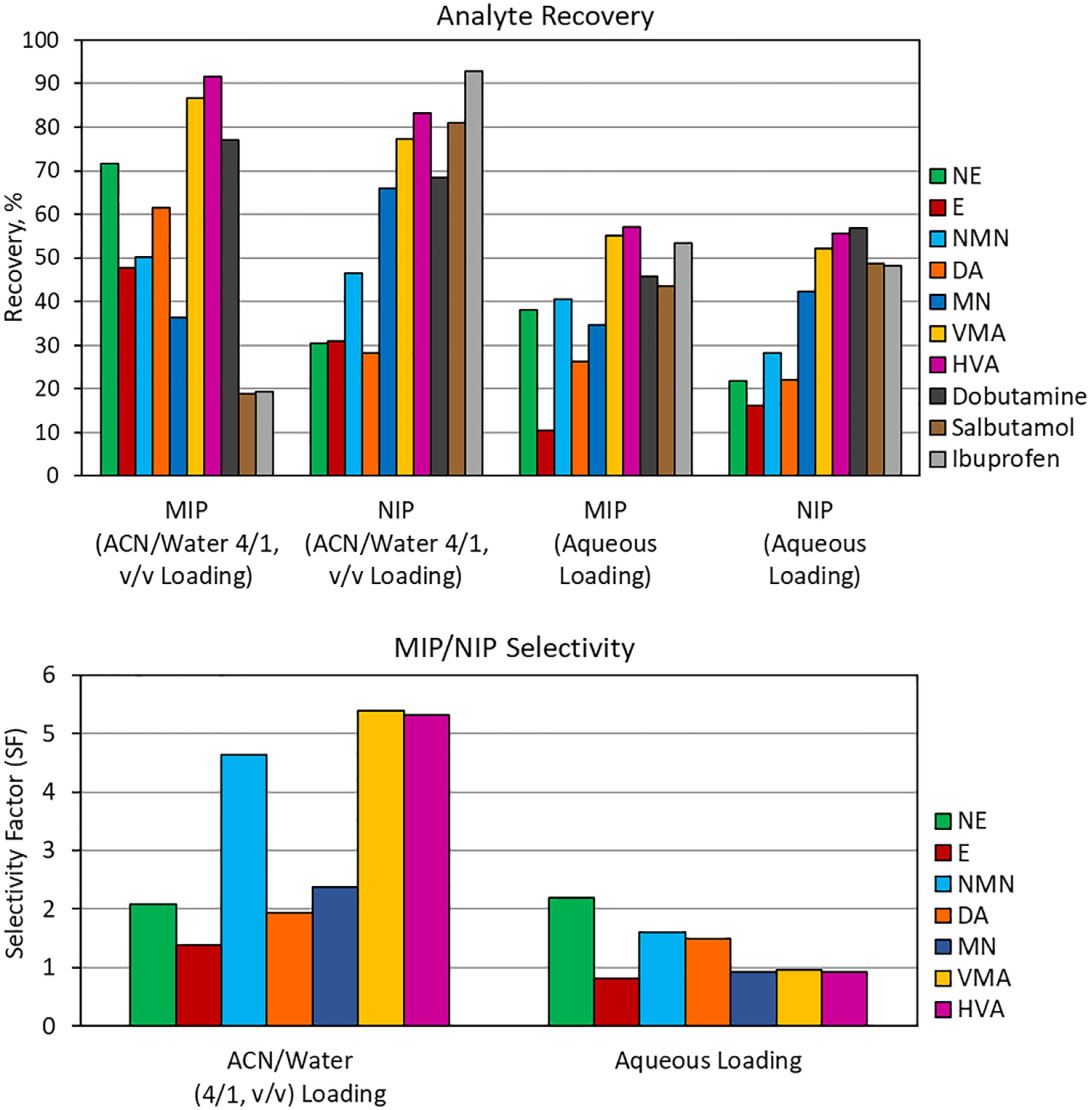
Analyte recovery and selectivity factor values for the optimized molecularly‐imprinted polymer (MIP) and the corresponding non‐imprinted control polymer (NIP) in acetonitrile/water 4/1 (v/v) loading and pure water loading (*n* = 1).

The first screening results showed that high MBAA‐to‐monomer ratios resulted in substantial loss of metanephrines (< 12% recovery), while high concentrations of monomer/template led to noticeable CA degradation, sorbent's gel‐like appearance, restricted solvent flow, and lack of selectivity (identical recovery on both NIPs and MIPs). Sorbent with 10:1 crosslinker‐to‐monomer ratio provided a balance between recovery and selectivity. Then, solvent system screening was carried out with different ratios of ACN, MeOH, and DMF. Two compositions, namely ACN/MeOH/DMF 5.67/1.33/1 (v/v) and ACN/MeOH 4/1 (v/v), offered medium‐to‐high recovery of CAs and metanephrines, while recovery of HVA and VMA exceeded 90%. Finally, different loadings of AIBN and V‐65 radical initiators were tested in both solvent systems. To better understand sorption selectivity, MIP/NIP pairs were tested by comparing recovery of CAs and CAMs to three sterically hindered reference medications that possess similar functional groups (dobutamine for CAs, salbutamol for metanephrines, and ibuprofen for HVA and VMA, respectively). Obtained recovery values were then used to calculate SF according to Equation 1. The sorption of sterically hindered medications is designed to be much more restricted compared to CAs and CAMs, thus acting as a reference. High recovery of these reference compounds means low SF values, indicating mostly non‐selective sorption (such as hydrogen bonding on the sorbent surface). It was determined that medications (except dobutamine) showed much stronger binding with NIP, suggesting the prevalence of non‐selective interactions. Selective binding is especially important for problematic analytes like MN and NMN, since *O*‐methylation lowers their polarity and increases steric hindrance compared to CAs. Radical initiator screening showed that SF values of MN and NMN were much lower for MIPs made with V‐65 (SF 1.1–1.9) compared to AIBN (SF 1.4–5.4), regardless of initiator loading or solvent system used. In the end, MIP made with 10:1 crosslinker‐to‐monomer ratio (133 mM MBAA; 13.3 mM of monomer/template complexes) and 16% AIBN loading (by solid weight) in ACN/MeOH/DMF (5.67/1.33/1, v/v) demonstrated the best compromise between analyte recovery, sorption selectivity, and permeability to solvents. It was therefore chosen for urine sample analysis and MISPE‐UHPLC‐MS/MS method validation. Recovery and SF values for the optimized MIP and the corresponding NIP are shown in Figure 3.

It was discovered that MIPs yielded higher analyte recovery and SF in ACN/water (4/1, v/v) compared to aqueous sample solutions (especially for E, NE, VMA, and HVA). Poor sorption selectivity in water is likely caused by a much more polar environment, which changes the shape of the imprinted cavities and/or disrupts selective analyte–sorbent interactions, leaving mostly non‐selective binding on the polymer surface.

### 3.2 Sorbent Physiochemical Characterization

#### 3.2.1 Fourier‐Transform Infrared Spectroscopy

Fourier‐transform infrared (FTIR) spectra of activated MIP and NIP are somewhat similar due to the abundance of MBAA (cross‐linker). Signals at 1108 and 1208, 1524, and 1644 cm^−1^ belong to C─N, C─O, and C═O bonds, respectively. Signal at 3294 cm^−1^ corresponds to tetraalkylammonium C─N bonds and O─H stretching from water/hydroxide trapped in the sorbent [31, 32]. MIP's signals at 1440, 1524, and 1644 cm^−1^ are more intense with a shoulder at 1562 cm^−1^, indicating the presence of unremoved template (DMPAA and HVAlc). Sorbent FTIR spectra are shown in Figure S1 (Supporting Information).

#### 3.2.2 Scanning Electron Microscopy

**FIGURE 4:**
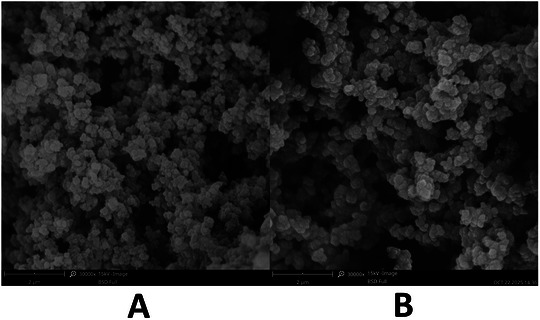
Scanning‐electron microscopy images of molecularly‐imprinted polymer (A) and non‐imprinted control polymer (B) at 30000× magnification.

SEM micrographs show the surface of MIP and NIP after OH^−^activation. Both sorbents consist of small particles (< 0.5 µm) that form spherical agglomerates. NIP's surface appears to be slightly smoother compared to MIP; the latter consists of smaller and more densely packed particles, likely caused by the influence of added templates. SEM micrographs are presented in Figure 4.

#### 3.2.3 Static Binding Experiments

**FIGURE 5:**
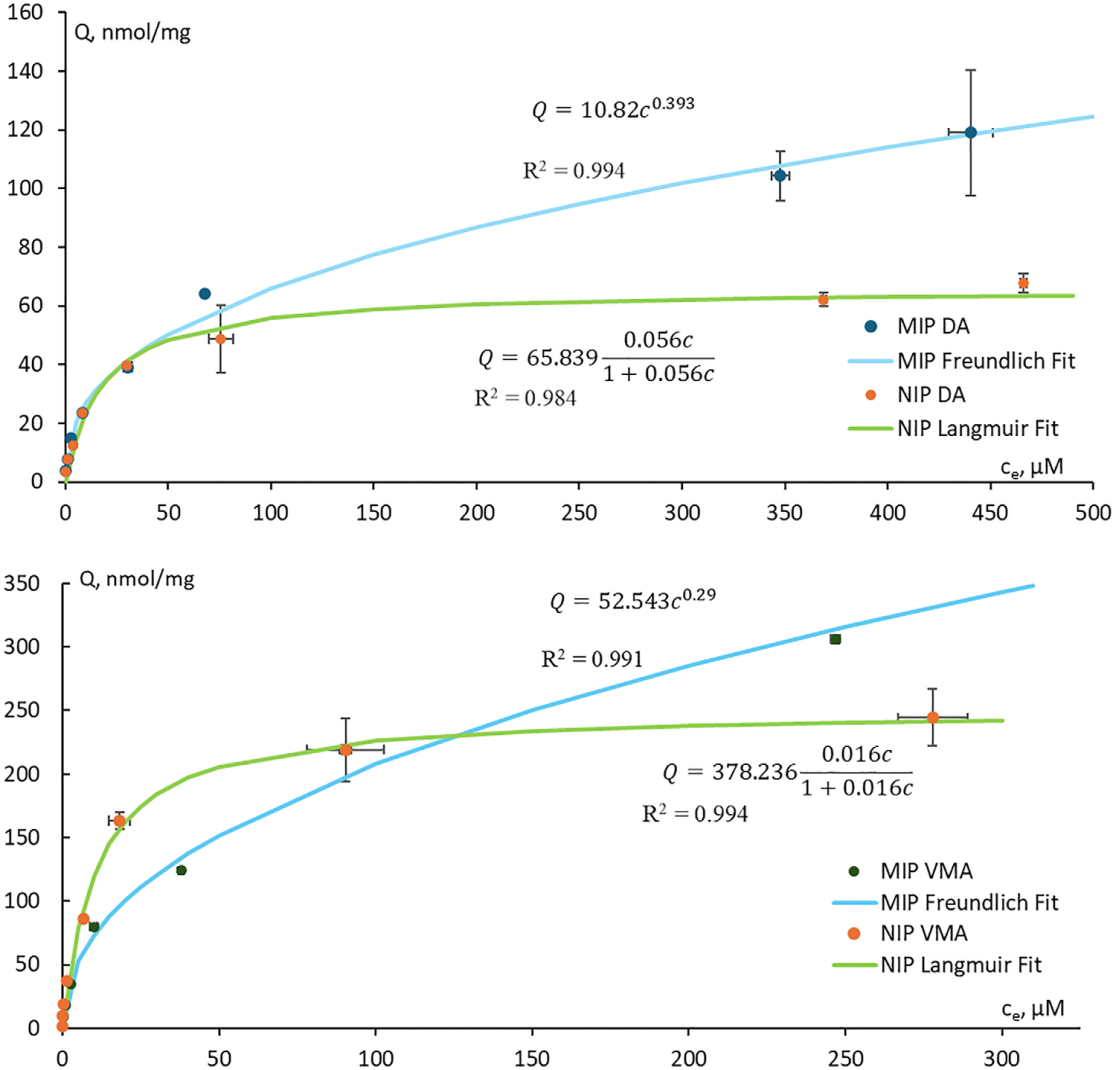
Fitted static binding isotherms of dopamine (top) and vanillylmandelic acid (bottom) on molecularly‐imprinted polymer (MIP) and non‐imprinted control polymer (NIP) with data point confidence intervals (95% certainty, *n* = 3).

The experiments were carried out in acetonitrile–methanol (4/1, v/v), as it is most similar to the solvent used during synthesis to minimize influence on sorbent's microscopic properties [30]. DA and VMA are representative analytes for CAs and CAMs, respectively. The Freundlich model provided the best fit for analyte binding on MIP, which is consistent with published literature: an increasingly heterogeneous surface is characteristic of non‐covalently imprinted polymers [23]. Analyte binding on NIP is best described by the Langmuir model. MIP demonstrates higher DA sorption capacity compared to NIP, especially at higher concentrations (124 mmol/mg and 71 mmol/mg at 500 µM, respectively). VMA initially binds better with NIP at low concentrations, but its sorption capacity effectively plateaus when analyte concentration exceeds 100 µM, meaning that NIP is likely described by non‐selective surface interactions that are quickly exhausted. Binding isotherms for DA and VMA on MIP and NIP are shown in Figure 5.

#### 3.2.4 Dynamic Binding Experiments

**FIGURE 6:**
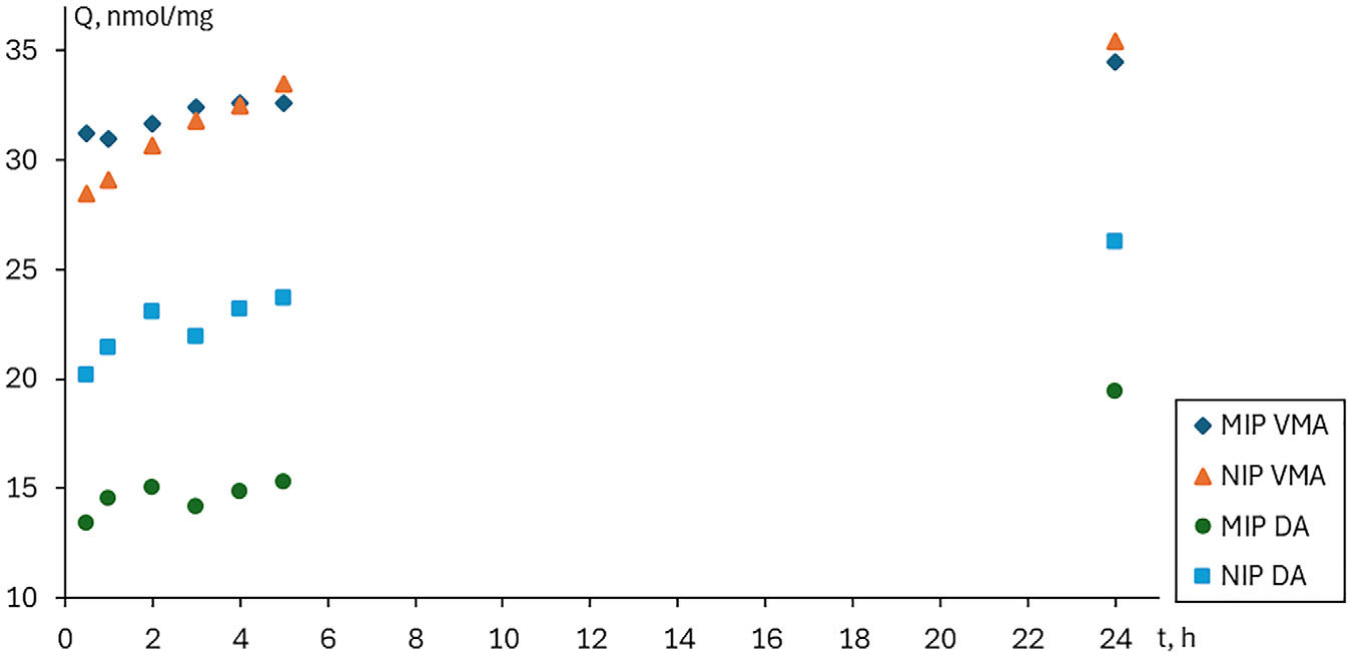
Dynamic binding curves of dopamine and vanillylmandelic acid on molecularly‐imprinted polymer (MIP) and non‐imprinted control polymer (NIP) at different time intervals (0.5–24 h).

The obtained dynamic binding curves (see Figure 6) suggest that most of the analyte binds within the first 30 min, with successive intervals providing a small increase over 24 h. The results suggest that both NIP and MIP provide more binding sites for VMA compared to DA, suggesting a larger number of non‐selective interactions on the surface (likely through hydrogen bonding between MBAA's nitrogen atoms and VMA's side‐chain functional groups).

#### 3.2.5 Brunauer–Emmett–Teller (BET) Surface Area and Pore Size Analysis

MIP sorbent's determined surface area is 49.098 m^2^/g with a total pore volume of 0.1141 cm^3^/g and an average pore diameter of 9.297 nm, showing that the MIP is a mesoporous material (2–50 nm pore size) [33]. NIP's results are 33.377 m^2^/g, 0.0624 cm^3^/g, and 7.480 nm, respectively. Higher pore volume, larger pore diameter and surface area typically indicate more developed pores and higher binding capacity. A newer batch of the same MIP formulation was analyzed as well, with similar results to the older batch: 45.964 m^2^/g, 0.1111 cm^3^/g, and 9.665 nm. Summary of the BET analyses is presented in Figure S2.

### 3.3 MISPE‐HPLC‐MS/MS Method Optimization With Urine Samples

The goal of this step was to ensure the highest possible recovery of CAs and CAMs while lowering the recovery of L‐DOPA, M‐DOPA, and 3‐OMD to minimize interference. NE and NE‐D6 initially demonstrated low retention and significant overlap with matrix components, which was resolved by using 100% water (Solvent A) at the start of the gradient. To minimize potential matrix interference, stronger MRM transitions were replaced with more selective alternatives during method development. MRM transitions for MN (198→180 *m*/*z*), NMN (184→166 *m*/*z*), and NE (170→152 *m*/*z*) were replaced by 180→148 *m*/*z*, 166→134 *m*/*z*, and 152→107 *m*/*z*, respectively (see Table 1). While signal intensities remained acceptable, analyte peak intensity and its separation from matrix signals improved significantly. Methanol was chosen over acetonitrile as the strong mobile phase, yielding acceptable retention times for all CAs. This also provided better separation of DA from L‐DOPA (RT 2.86 min vs. 3.00 min) and VMA from methyldopa (RT 4.95 min vs. 5.25 min). The combined MRM chromatograms of an extracted pre‐spiked urine sample are shown in Figure S3.

Optimization was conducted by spiking urine samples before and after SPE. Analyte recovery was calculated by dividing the pre‐spike peak area by the corresponding post‐spike area. To account for the matrix effect, each analyte signal was normalized against its deuterated reference (added to samples before SPE). 60 mg MIP loading provided the best recovery values while still retaining adequate solvent flow. While urine samples typically require neutralization of excess HCl before SPE (especially for ion exchange and mixed‐mode cartridges) [34], our OH^−^ ‐activated sorbent successfully neutralized acidified urine (pH 2) in situ, bringing its pH to 8–9. Several SPE sample loading, washing, and elution phases were tested for the best recovery values.

For sample loading, different concentrations of AsA (used as an antioxidant) in water and methanol were tested. ACN was the loading solution baseline. Washing and eluting solutions were the same as in MIP optimization studies. As VMA tended to bind strongly with 60 mg of MIP, the volume of eluting solvent was increased from 0.5 mL to 1 mL to avoid substantial VMA loss. Aqueous loading solution showed lower recoveries for CAs, while acetonitrile could not dissolve AsA. The presence of AsA in methanol improved recoveries for CA and MNs in all tested concentrations (1, 2, 5, and 10 mM). A 5 mM AsA solution in MeOH provided optimal results (higher concentrations did not show any improvement). While the 5 mM AsA solution (MeOH) offered slightly lower results for VMA and HVA compared to ACN, it improved the recovery of NE and NMN and was chosen for further optimization studies.

For washing solution optimization, ACN/MeOH (1/1, v/v), methanol, pure water, and ACN/water (4/1, v/v) were used. The ACN/MeOH baseline mix and water provided medium to high results, while ACN/water yielded the lowest CA recoveries, likely due to high polarity and/or analyte degradation. Pure methanol gave satisfactory results. Several pH‐adjusting additives in methanol were then tested: phosphoric acid, AmForm, ammonium acetate, and AmBic (25 mM each). Methanol/water solution (95/5, v/v) was used in place of pure methanol to improve the solubility of the salts. AmBic solution showed improvements over pure methanol, likely caused by its mildly basic properties affecting analyte binding. Different concentrations of AmBic were tested (25–100 mM), with 50 mM offering high and consistent recovery values (especially for MN and NMN) while still lowering the presence of interfering medications. Higher AmBic concentrations lowered the recoveries, likely due to excessive ionic strength.

**FIGURE 7:**
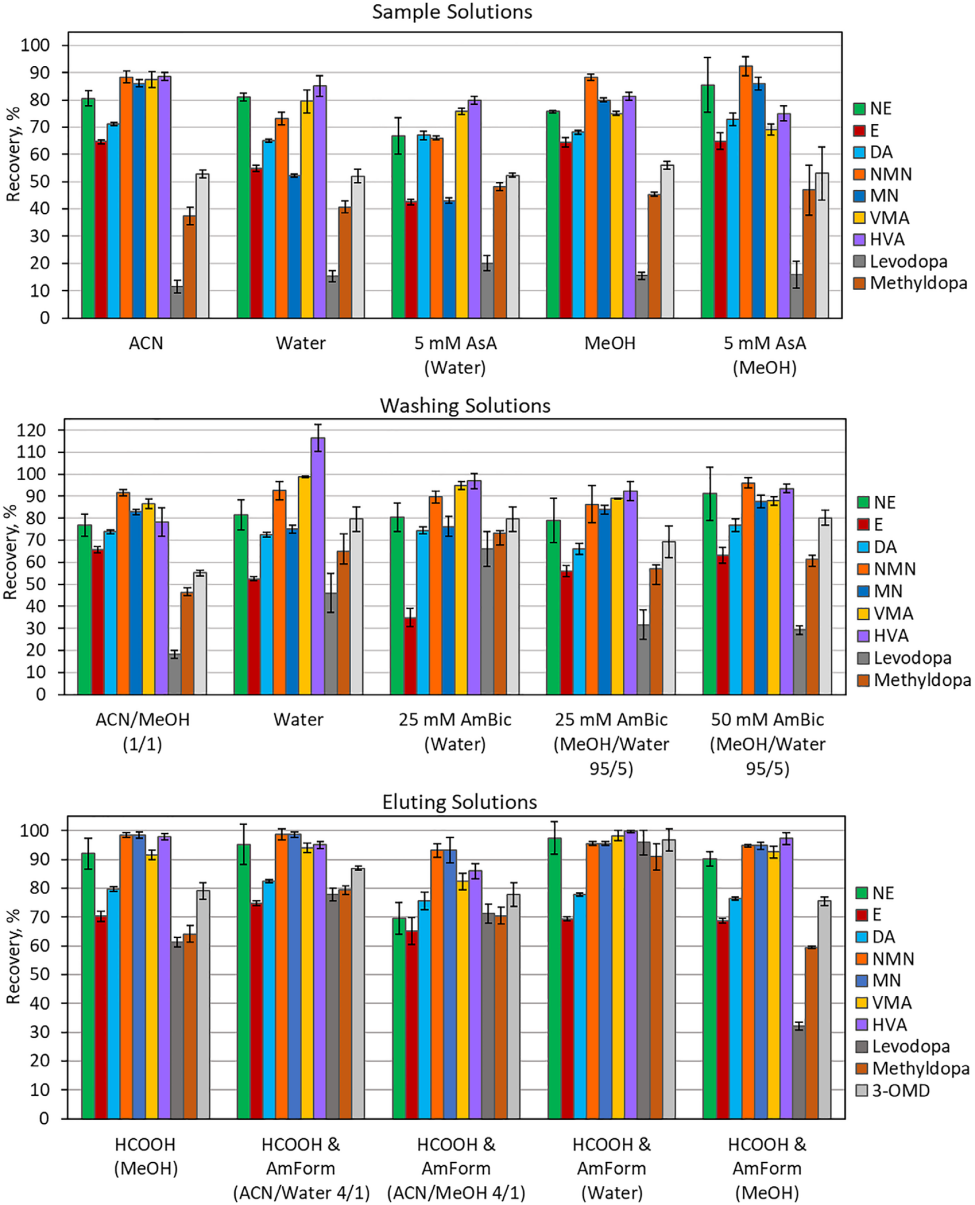
Analyte recovery values in selected sample, washing and eluting solutions (standard deviation, *n* = 3).

Solutions of formic acid (HCOOH) and AmForm in methanol, water, MeOH/water (4/1, v/v) and ACN/water (4/1, v/v) were used for elution optimization experiments. The baseline eluent used in all earlier tests (2% formic acid and 25 mM AmForm in MeOH) yielded the best overall results compared to other candidates, with recovery exceeding 69% for all seven target analytes (with NMN, MN, HVA, and VMA exceeding 90%). This final procedure was used for validation. L‐DOPA's recovery remained fairly low at 32%, while methyldopa showed moderate results at 60%. 3‐OMD had the largest recovery (70%) among interferents, likely due to its closer structural similarity to the templates. Despite much larger concentrations of these interferents in the spiked sample compared to CAs (0.20 mg/L vs. 3.7–30.6 µg/L of CAs), the sorbent maintained high recoveries for the analytes, which suggests the presence of molecular recognition. Recovery values for the selected sample, washing, and elution solutions are shown in Figure 7.

It was observed that a large presence of water during washing and elution leads to higher recovery levels of L‐DOPA, methyldopa, and 3‐OMD compared to organic solvents, possibly caused by water disrupting selective interactions and/or causing sorbent swelling. To determine the fate of these interferents during SPE in the organic phase, loading and washing fractions were analyzed. Approximately 1/3 of methyldopa and 3‐OMD elute from the cartridge during washing, whereas L‐DOPA is not detected in sample loading or washing solutions, suggesting that it binds strongly with the sorbent (stronger than VMA) and does not elute.

### 3.4 MISPE‐HPLC‐MS/MS Method Validation

The developed method was validated by testing its repeatability, intermediate precision, trueness, linearity, LOD, and LOQ. Data processing was carried out using Agilent QQQ Quantitative Analysis Software (Quant‐My‐Way).

#### 3.4.1 Repeatability, Intermediate Precision, and Trueness

Chromsystems certified QCs were used to determine repeatability at low and high concentration levels, with five replicates for each level analyzed within the same batch. All seven analytes yielded good repeatability with RSD values below 10%. Trueness and intermediate precision were determined at low and high concentration levels, with 10 replicates for each level in five separate batches (two replicates per batch). At low concentrations, only HVA and VMA slightly exceeded the ±15% nominal value threshold. At higher levels, all determined concentrations stayed within acceptable trueness values. On both levels, measured VMA concentrations were systematically lower, while the opposite was observed for HVA. Since the calibrators (Eureka) and QCs (Chromsystems) used were from two different manufacturers, sample non‐commutability is possible, though all obtained calibrations fall within the acceptable ranges. Eureka QCs were not available at the time. All measurements’ RSD values are below 10%, indicating good precision. Repeatability, trueness, and precision results are presented in Table 2.

**TABLE 2:** Method's trueness, repeatability, and intermediate precision.

Analyte	Stated concentration	Repeatability		Intermediate precision	
		Measured ± SD (*n* = 5)	RSD, % (*n* = 5)	Measured ± SD (*n* = 10)	Trueness ± RSD, % (*n* = 10)
NE (µg/L)	84.1	83,8 ± 4.5	5.4	87.1 ± 7.5	103.6 ± 8.6
	287	306 ± 7	2.4	294 ± 20	102.6 ± 6.8
E (µg/L)	19.3	17.1 ± 0.3	1.8	18.0 ± 0.6	93.2 ± 3.2
	164	151 ± 4	2.5	161 ± 4.8	98.3 ± 3.0
NMN (µg/L)	76.9	79.2 ± 3.9	4.9	78.8 ± 3.3	102.4 ± 4.2
	566	674 ± 20	2.9	587 ± 35	103.7 ± 5.9
DA (µg/L)	359	377 ± 7	1.9	372 ± 12	103.8 ± 3.1
	727	758 ± 10	1.4	769 ± 25	105.7 ± 3.3
MN (µg/L)	77.5	82.9 ± 1.3	1.5	84.5 ± 3.3	109.1 ± 3.9
	697	779 ± 5	0.70	791 ± 26	113.4 ± 3.3
VMA (mg/L)	5.70	4.67 ± 0.15	3.4	4.67 ± 0.17	81.9 ± 3.7
	38.1	31.1 ± 0.7	2.1	32.4 ± 1.0	85.0 ± 2.9
HVA (mg/L)	6.42	7.27 ± 0.16	2.1	7.43 ± 0.36	115.7 ± 4.8
	57.5	60.4 ± 0.7	1.2	65.5 ± 1.9	113.9 ± 3.1

#### 3.4.2 Linearity, LOD, and LOQ

Linearity was estimated in five MISPE batches using a 6‐point certified Eureka calibration kit. All seven analytes demonstrated good linearity with determination coefficients (*R* ^2^) exceeding 0.99 and accuracy staying within ±15% of nominal values with 1/*x* weighing factor. All analytes in Level 1 Calibrator had a satisfactory SNR ratio of at least 10, with all measured concentrations falling within ±20% of the nominal value provided by the manufacturer. Obtained LOQ values are comparable to the lower end of the reference intervals used for PHEO assays, while the reported linear range is higher than published upper reference limits for PHEO, making the method suitable for bioanalysis [35, 36]. Linearity, LOD, and LOQ data are summarized in Table 3.

**TABLE 3:** Method's linearity, limits of detection, and limits of quantitation.

Analyte	LOD	LOQ	Linear range	Slope ± SE	Intercept ± SE	*R* ^2^	Accuracy range, %
NE (µg/L)	3.3	6.5	12.8–265.6	(1.54 ± 0.03) × 10^−2^	(1.2 ± 1.9) × 10^−2^	0.992	91.6–109.0
E (µg/L)	0.4	0.7	1.8–97.9	(3.95 ± 0.05) × 10^−3^	(0.9 ± 0.8) × 10^−3^	0.996	96.8–105.1
NMN (µg/L)	0.2	1.5	25.6–772.5	(2.54 ± 0.03) × 10^−3^	(0.1 ± 0.5) × 10^−2^	0.997	97.3–101.7
DA (µg/L)	1.0	6.5	40.8–1345.8	(1.711 ± 0.016) × 10^−3^	(2.8 ± 0.5) × 10^−2^	0.997	98.3–104.1
MN (µg/L)	0.3	1.3	22.4–883.7	(4.38 ± 0.05) × 10^−2^	(3.2 ± 0.9) × 10^−1^	0.997	93.0–105.8
VMA (mg/L)	0.04	0.12	0.7–19.0	(2.12 ± 0.03) × 10^−1^	(1.0 ± 1.4) × 10^−2^	0.995	94.6–105.2
HVA (mg/L)	0.06	0.2	1.1–26.0	(9.01 ± 0.17) × 10^−2^	(6.5 ± 1.1) × 10^−2^	0.991	93.4–104.9

### 3.5 MIP Regeneration and Comparison to Commercial SPE Sorbents

**FIGURE 8:**
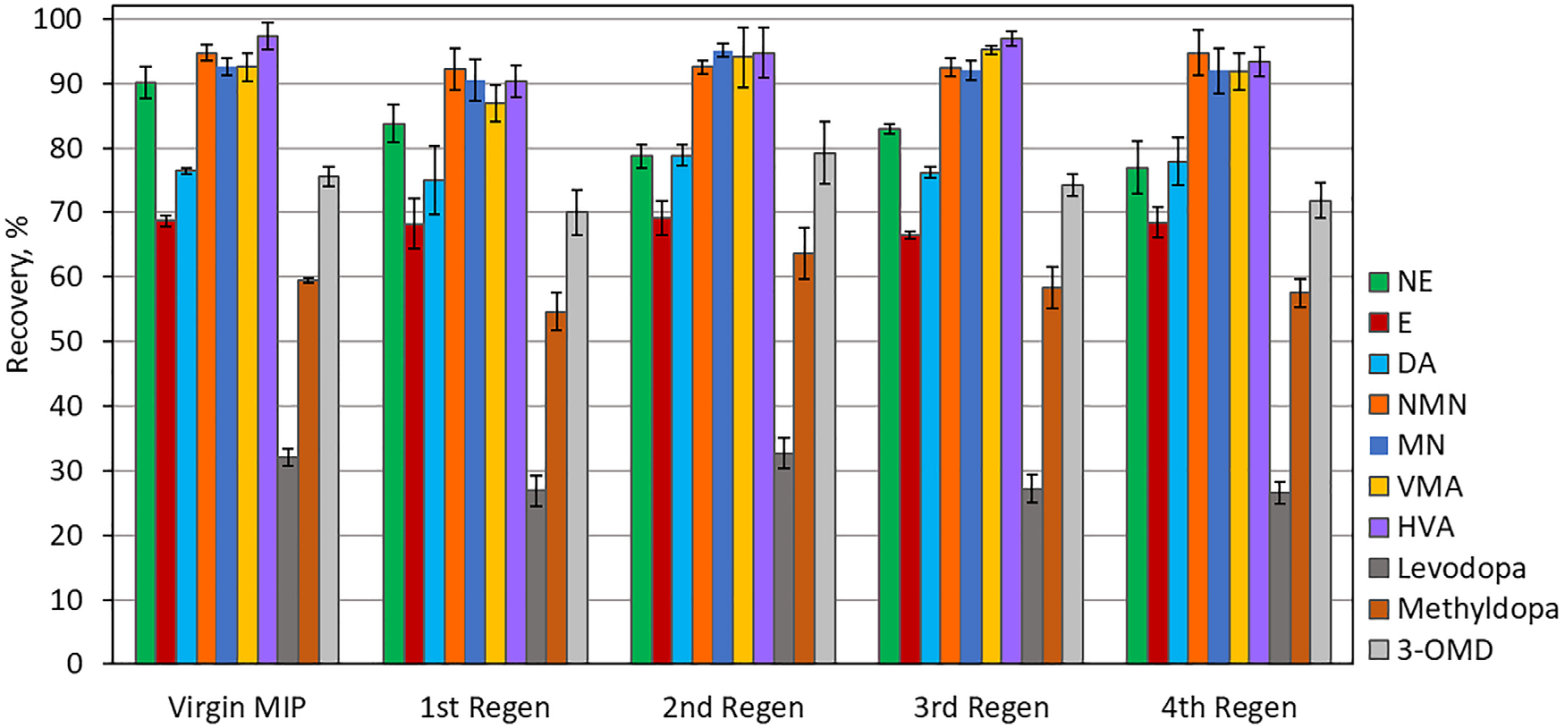
Analyte recovery values during sorbent regeneration studies (standard deviation, *n* = 3).

To determine MIP's reuse potential, spent SPE cartridges were repeatedly regenerated with a caustic solution and subjected to the optimized MISPE procedure, with four reuse cycles in total. Only NE demonstrated a slight recovery decrease (90%–77%). However, this influence on NE quantitation is mostly negated by using an isotopically labeled internal standard. All other analytes showed no performance degradation, meaning that the sorbent can be successfully recycled. Results of MIP regeneration are presented in Figure 8.

**FIGURE 9:**
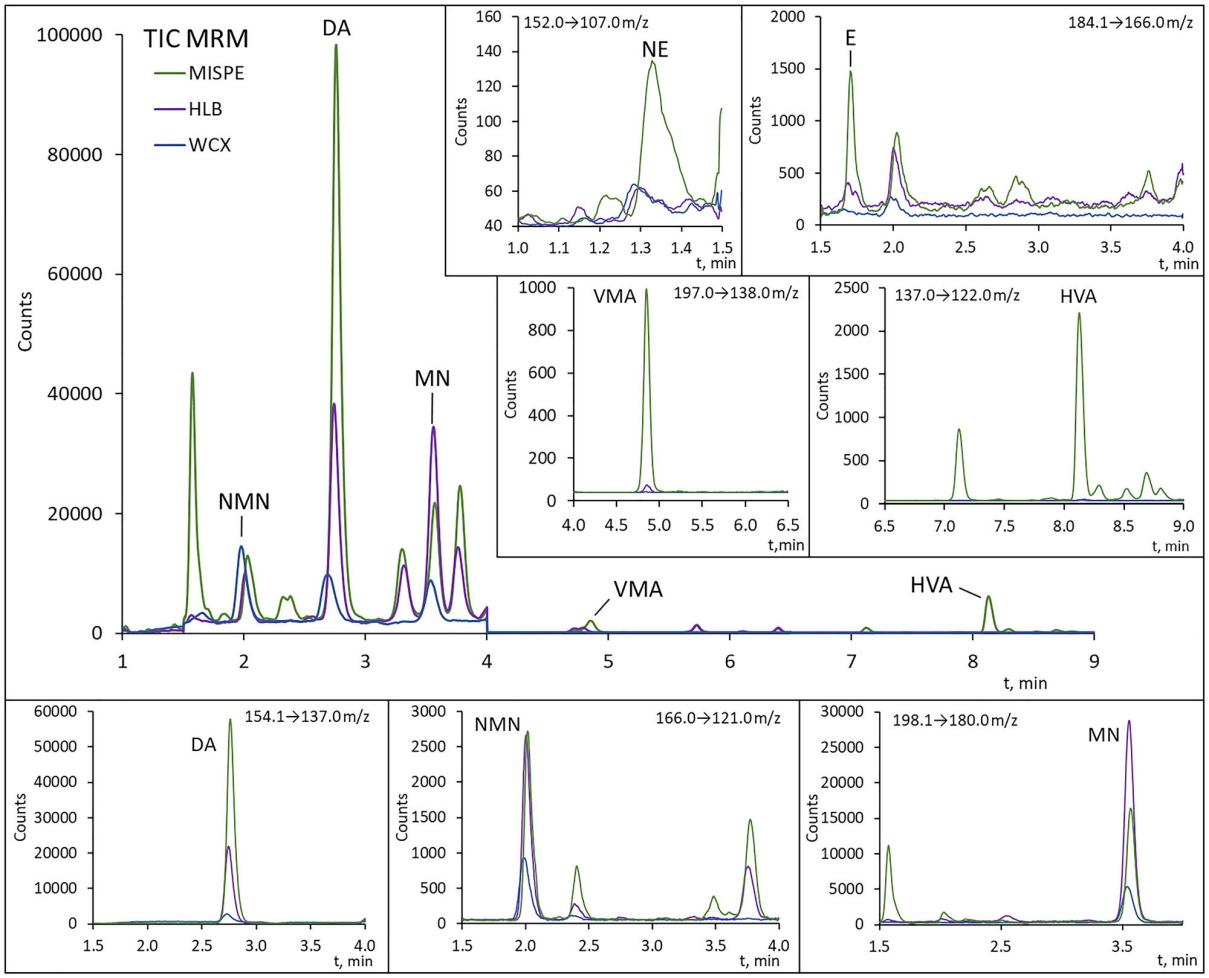
Total ion current multiple reaction monitoring (TIC MRM) and analyte multiple reaction monitoring (MRM) chromatograms of non‐spiked pooled urine after molecularly‐imprinted solid‐phase extraction (MISPE, 10× dilution), hydrophilic‐lipophilic balance solid‐phase extraction (HLB, 2× dilution), and weak cation exchange solid‐phase extraction (WCX, 2.5× dilution).

The synthesized MIP was also compared to commercially available WCX and HLB (Waters Oasis HLB) SPE cartridges. Non‐spiked pooled urine was used to visually assess the matrix influence on endogenous analytes (in TIC MRM mode and in individual MRM transitions). Urine dilution factors were the same as in the original protocols (10× for MISPE, 2× for HLB, and 2.5× for WCX). Despite having larger dilution, DA, MN, NMN, HVA, and VMA can be clearly seen in MISPE's TIC MRM. Most importantly, MISPE allows to identify NE's and E's MRM signals, which are located in the challenging ion suppression region, where polar urine interferents are located. Meanwhile, NE and E are effectively suppressed for HLB and WCX. MISPE also demonstrated its dual‐recognition ability, as it successfully retained VMA and HVA (HLB produced a much weaker signal, while WCX is incompatible with acidic analytes). TIC MRM and individual MRM transition chromatograms are shown in Figure 9.

## 4 Conclusions

This paper detailed the synthesis and use of novel non‐covalent MIP sorbent utilizing strong anion exchange for simultaneous isolation of CAs, MNs, VMA, and HVA from acidified human urine in a single SPE run without additional pH readjustment. The method avoids substantial analyte decomposition, produces stronger CA signals compared to commercial SPE phases, and maintains molecular recognition ability in the presence of structurally similar interfering medications in high concentrations. Sorbent synthesis requires only basic equipment and widely available chemicals. The imprinted polymer can also be reused at least four times with satisfactory recoveries (up to 97%) and with no performance degradation, reducing reagent and labor costs. The validated MISPE‐HPLC‐MS/MS method is compatible with analyte reference concentrations used for PHEO assays, demonstrating its potential for use in bioanalysis.

## Author Contributions

**Artūrs Šilaks**: investigation, formal analysis, visualization, supervision, validation, writing – original draft, writing – review and editing. **Antons Podjava**: methodology, resources, supervision, project administration, funding acquisition, writing – review and editing. **Laura Bernāte**: methodology, investigation, formal analysis. **Vladlens Grebnevs**: investigation, formal analysis. **Artur Maciej**: Investigation.

## Ethics Statement

The study protocol was reviewed and approved by the Ethics Committee for Life Sciences and Medical Research of the University of Latvia (Approval No. 13–22/20).

## Conflicts of Interest

The authors declare no conflicts of interest.

## Supporting information




**Supporting File**: jssc70467‐sup‐0001‐SuppMat.docx.

## Data Availability

Supporting Information are available online as a separate document. Raw experimental data will be made available upon reasonable request from the corresponding author.
