# Supplementary material for: Novel Strongly Basic Molecularly Imprinted Solid‐Phase Extraction Sorbent for Simultaneous Determination of Catecholamines and Their Metabolites in Urine
Source: J Sep Sci. 2026 Jun 14;49(6):e70467. doi: 10.1002/jssc.70467 (PMC13266282; doi:10.1002/jssc.70467)
Supplement: Supplementary file 1 — Supporting File: jssc70467‐sup‐0001‐SuppMat.docx. [file JSSC-49-e70467-s001.docx]

**Supplementary Materials**

**Novel Strongly Basic Molecularly-Imprinted Solid-Phase Extraction Sorbent for Simultaneous Determination of Catecholamines and Their Metabolites in Urine**

**Artūrs Šilaks^1*^, Antons Podjava^1^, Laura Bernāte^1^, Vladlens Grebnevs^1,2^ and Artur Maciej^2^**

^1^ University of Latvia, Faculty of Medicine and Life Sciences, Jelgavas Str. 1, Riga LV-1004, Latvia; an-tons.podjava@lu.lv; laurabernate19@gmail.com

^2^ Silesian University of Technology, Faculty of Chemistry, B. Krzywoustego Str. 6, Gliwice 44-100, Poland; vladlens.grebnevs@polsl.pl; artur.maciej@polsl.pl

* Correspondence: arturs.silaks@lu.lv; Tel.: +371 26144666

**Table of Contents**

[1. Reagents and solvents 3](#_Toc228844314)

[2. Characterization of functional monomer/template complexes 4](#_Toc228844315)

[3. HPLC/PDA analysis for polymerization monitoring and MIP optimization studies 5](#_Toc228844316)

[4. Sorbent physiochemical characterization 6](#_Toc228844317)

[4.1. Fourier-transform infrared spectroscopy (FTIR) 6](#_Toc228844318)

[4.2. HPLC/PDA analysis of binding experiments 7](#_Toc228844319)

[4.3. Scanning electron microscopy (SEM) 7](#_Toc228844320)

[4.4. Brunauer–Emmett–Teller (BET) surface area and pore size analysis 7](#_Toc228844321)

[5. MISPE-HPLC-MS/MS method optimization and validation 9](#_Toc228844322)

# **1. Reagents and solvents**

**Catecholamine & drug analytical standards:** (±)-Norepinephrine (NE) (+)-bitartrate, *D,L*-metanephrine (MN) HCl, 3-methoxy-L-tyrosine (3-*O*-methyldopa, 3-OMD) monohydrate and salbutamol (≥98%) were procured from Sigma-Aldrich (St. Louis, USA). (–)-Epinephrine (E) (+)-bitartrate was delivered by Acros Organics (Fair Lawn, USA). Levodopa (L-DOPA) (Pharmaceutical CRM), dopamine (DA) HCl, *D,L*-normetanephrine (NMN) HCl (≥98%) and ibuprofen (≥98%, GC quality) were obtained from Merck (Darmstadt, Germany). Homovanillic acid (HVA) and vanillylmandelic acid (VMA) were bought from Fluorochem (Hadfield, UK). Dobutamine HCl (British Pharmacopeia CRS) and methyldopa (British Pharmacopeia CRS) were sourced from the British Pharmacopeia Commission Laboratory (Teddington, UK).

**Deuterated internal standards:** NE-D6, E-D6, DA-D4 HCl, NMN-D3 HCl and MN-D3 HCl (100 μg/mL solution in methanol each, free base concentration) were procured from Cerilliant/Sigma-Aldrich (St. Louis, USA). HVA-D5 (100 mg/L in methanol) and VMA-D3 (100 mg/L in methanol) were purchased from Merck (Darmstadt, Germany).

**Reagents for sorbent synthesis:** 2,2’-Azobisisobutyronitrile (AIBN) was bought from Glentham Life Sciences (Corsham, UK). 2,2’-Azobis(2,4-dimethylvaleronitrile) (V-65) was purchased from Combi Blocks (San Diego, USA). 3,4-Dimethoxyphenylacetic acid (homoveratric acid, DMPAA, 99%), lithium aluminum hydride (4.0M solution in ether) and Ambersep 900 OH anion exchange resin were provided by Acros Organics (Fair Lawn, USA). 4-Vinylbenzyl chloride (90%, technical grade), tetrahydrofuran (99.5%, AcroSeal Extra Dry) and trimethylamine (4.2M solution in ethanol, pure) were purchased from ThermoScientific (Waltham, USA). *N,N’*-Methylenebisacrylamide (MBAA, 99%) was provided by Sigma-Aldrich (St. Louis, USA).

**Other reagents:** Ammonium bicarbonate and ascorbic acid (ACS purity) were purchased from Enola SIA (Riga, Latvia). 25% aqueous ammonium hydroxide solution (Suprapur for HPLC) and concentrated hydrochloric acid (36.5-38.0%, biology grade) were provided by Sigma-Aldrich/MilliporeSigma (St. Louis, USA). Formic acid (purum), sodium bicarbonate (chemically pure), sodium chloride (purum), anhydrous sodium sulfate (chemically pure), sodium sulfate decahydrate (purum) and sodium hydroxide (pro analysis) were provided by reliable local suppliers.

**Solvents:** LMCS-grade formic acid (HCOOH) was supplied by VWR Chemicals/Avantor (Radnor, USA). *N,N*-Dimethylformamide (DMF) was bought from Carl Roth GmbH (Karlsruhe, Germany). Ethyl acetate (HPLC grade), petroleum ether (40-60 °C, reagent grade), methanol (HPLC Grade) and acetonitrile (HPLC Grade) were bought from Fisher Scientific (Loughborough, UK). LC-MS Chromasolv methanol and LC-MS Chromasolv acetonitrile were sourced from Honeywell (Muskegon, USA). Grade 1 deionized water (<0.05 μS/cm) was made using Adrona B30 Purification System (Riga, Latvia).

# **2. Characterization of functional monomer/template complexes**

In addition to ^1^H and ^13^C NMR, the identity of VBTMA-DMPAA and VBTMA-HVAlc was further verified using HRMS and FTIR. IR spectra were obtained using Perkin-Elmer Frontier FT-IR/FIR in Attenuated Reflectance Recording (ATR) mode in 550-4000 cm^−1^ range with data acquisition rate of 16 scans/s. ^1^H-NMR (300 MHz) and ^13^C-NMR (75 MHz) spectra were recorded in CD_3_OD or CDCl_3_ using Bruker Fourier 300 spectrometer and analyzed using MestReNova 12. Chemical shifts are expressed in parts per million (ppm).

**(4-Vinylbenzyl)trimethylammonium-homoveratric acid anion salt (VBTMA-DMPAA):**

IS (thin layer, cm^−1^): 3367 (O-H, broad), 3031 (C-N), 2939 (C-N), 1571 (C=O or C=C), 1368 (C-O), 1231 (C-O or C-N), 1146 (C-O), 1025 (C-O), 861 (arom. C-H).

HRMS ESI+ (*m/z*): [C_12_H_18_N]^+^ calculated: 176.1434. Reported: 176.1434. [C_10_H_12_O_4_+H]^+^ Calculated: 197,0808. Reported: 197.0808.

HRMS ESI− (*m/z*): [C_10_H_11_O_4_−H]^−^ calculated: 195,0663. Reported: 195,0664.

**(4-Vinylbenzyl)trimethylammonium-homovanillyl alcohol anion salt (VBTMA-HVAlc):**

IS (thin layer, cm^−1^): 3248 (O-H, broad), 3030 (C-N), 2938 (C-N), 1646 (C=C), 1486 (C-O)), 1284 (C-O or C-N), 1042 (C-O), 834 (arom. C-H).

HRMS ESI+ (*m/z*): [C_12_H_18_N]^+^ calculated: 176.1434. Reported: 176.1439.

HRMS ESI− (*m/z*): [C_9_H_11_O_3_]^−^ calculated: 167.0714. Reported: unstable under HRMS conditions.

# **3. HPLC/PDA analysis for polymerization monitoring and MIP optimization studies**

Polymerization reactions were monitored with Shimadzu Prominence HPLC/PDA System (LC-20AD pump, DGU-20A3 degasser, SIL-20A autosampler, CT0-10ASVP oven, SPD-M20A DAD and Waters XTerra C18 (3 µm, 2.1×150 mm, 30 °C oven temperature). The mobile phases were 0.1% (v/v) formic acid in water (A) and pure acetonitrile (C). The analyses were run in gradient mode: solvent C was increased from 10 to 90% in 10 min, then kept at 90% C for 5 minutes. Flow rate was constant at 0.2 ml/min.

Samples from MIP optimization studies were analyzed using the same Shimadzu Prominence HPLC System but with Atlantis T3 column (3 µm, 2.1×150 mm). The mobile phases were 0.1% (v/v) formic acid in water (A), 0.1% (v/v) formic acid in methanol (B) and 0.1% (v/v) formic acid in acetonitrile (C). The data were acquired in 210-400 nm range and analyzed using Shimadzu LCSolution. The analyses were run in gradient mode: B was first changed from 0 to 20% in 6 min and then held for 14 minutes. Then B was set to 0, while C was set to 20%. Then C was increased to 60% C in 6 mins and held constant for 20 mins. The oven was set at 30 °C, flow rate was 0.2 ml/min.

# **4. Sorbent physiochemical characterization**

## **4.1. Fourier-transform infrared spectroscopy (FTIR)**

IR spectra were obtained using Perkin-Elmer Frontier FT-IR/FIR in Attenuated Reflectance Recording (ATR) mode in 550-4000 cm^−1^ range with data acquisition rate of 16 scans/s. Sorbent suspension in methanol (20 mg/mL) was applied to the crystal and then analyzed neat after waiting for solvent to evaporate.





**Figure S1.** Fourier-transform infrared spectra for hydroxide-activated molecularly imprinted polymeric sorbent (MIP, solid line) and activated non-imprinted control sorbent (NIP, dashed line). The insert (bottom left) shows signals in 800-1700 cm^−1^ region in more detail

## **4.2. HPLC/PDA analysis of binding experiments**

Static and dynamic binding sample solutions were analyzed using Agilent 1290 Infinity II System, which consisted of G7104A flexible quaternary pump, G7167B multisampler, G7116B multicolumn thermostat, G7117B diode array detector and Waters Atlantis T3 column (3 µm, 2.1×150 mm). The mobile phases were 0.1% (v/v) formic acid in water (A) and 0.1% (v/v) formic acid in acetonitrile (C). Both the calibrations and binding analyses were run for 14 mins in isocratic mode at 97% A at 30 °C. Mobile phase flow rate was 0.2 ml/min. VMA and DA detection wavelength was 278 nm. The data was analyzed using Agilent MassHunter.

## **4.3. Scanning electron microscopy (SEM)**

SEM was performed with Phenom ProX microscope (ThermoFisher Scientific, the Netherlands). Imaging was carried out at an accelerating voltage of 15 kV. Micrographs were collected at a range of suitable magnifications to identify distinct microstructural features. For SEM observation, the dry sorbent powder was gently applied as is onto conductive carbon tape mounted on a standard SEM stub. To reduce charging effects and enhance image stability, the mounted powders were gold-coated by sputter deposition in an ionized argon atmosphere using Sputter Coater 108 Auto (Cressington Scientific Instruments, Watford, UK) for 180 s.

## **4.4. Brunauer–Emmett–Teller (BET) surface area and pore size analysis**

Quantachrome NOVA 4200e Surface Area and Pore Size Analyzer (Anton Paar Inc., Ashland, USA) was used. Nitrogen gas adsorption–desorption isotherms were obtained with 1 cm^3^ of sample at liquid nitrogen temperature with the following relative pressure P/P0 range: 0.005–0.99–0.1 (analysis time: 1010.7 min). Specific surface area of the sorbents was determined using BET Method, with P/P0 interval of 0.005–0.3. Data acquisition and analysis was performed using Quantachrome NovaWin software. Summary of surface area and pore size analysis results are presented in Figure S2.


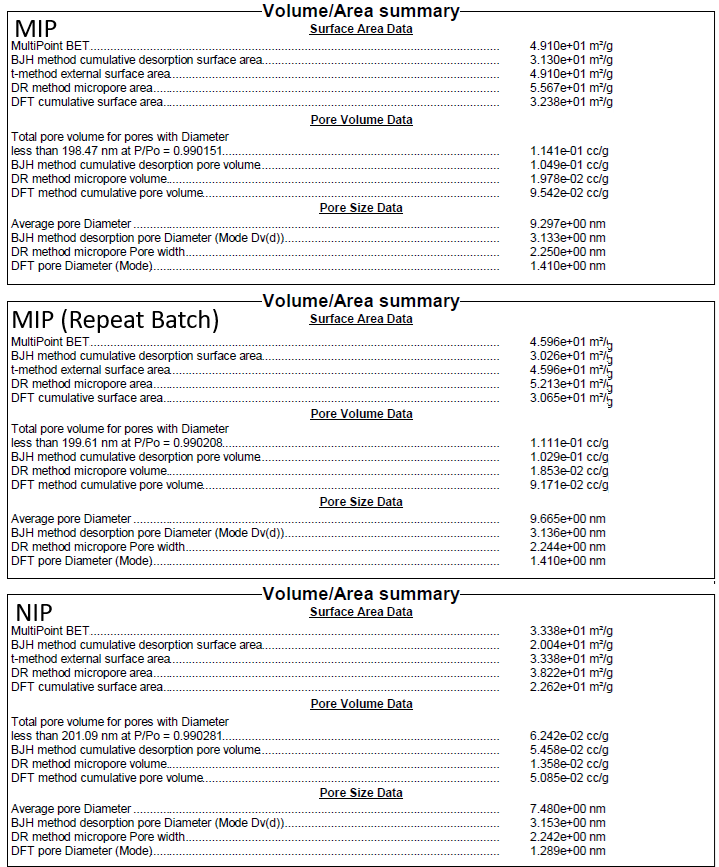


**Figure S2.** Surface area and pore size analysis summary for the optimized molecularly imprinted polymeric sorbent (MIP), repeat batch of the same imprinted sorbent formulation and the non-imprinted control polymer (NIP)

# **5. MISPE-HPLC-MS/MS method optimization and validation**

**Table S1.** Concentrations of catecholamines, free metanephrines, vanillylmandelic acid and homovanillic acid in Eureka calibrators

| **Level** | **NE** | **E** | **DA** | **NMN** | **MN** | **VMA** | **HVA** |
| --- | --- | --- | --- | --- | --- | --- | --- |
|  | **ug/L** | | | | | **mg/L** | |
| 0 | 6.5 | 0.7 | 32.5 | 7.4 | 2.5 | 0.6 | 1.0 |
| 1 | 12.8 | 1.8 | 40.8 | 25.6 | 22.4 | 0.7 | 1.1 |
| 2 | 43.1 | 7.7 | 189.1 | 102.4 | 108.5 | 3.0 | 4.0 |
| 3 | 59.0 | 25.4 | 344.1 | 195.4 | 216.5 | 5.4 | 6.8 |
| 4 | 116.6 | 48.2 | 657.0 | 265.6 | 435.0 | 9.0 | 13.2 |
| 5 | 175.4 | 73.0 | 966.9 | 573.5 | 668.4 | 14.5 | 19.0 |
| 6 | 265.6 | 97.9 | 1345.8 | 772.5 | 883.7 | 19.0 | 26.0 |

**Table S2.** Target concentrations of catecholamines, free metanephrines, vanillylmandelic acid and homovanillic acid in Chromsystems quality control standards

| **Level** | **NE** | **E** | **DA** | **NMN** | **MN** | **VMA** | **HVA** |
| --- | --- | --- | --- | --- | --- | --- | --- |
|  | **ug/L** | | | | | **mg/L** | |
| 1 | 84.1 | 19.3 | 76.9 | 359 | 77.5 | 5.70 | 6.42 |
| 2 | 287 | 164 | 566 | 727 | 697 | 38.1 | 57.5 |


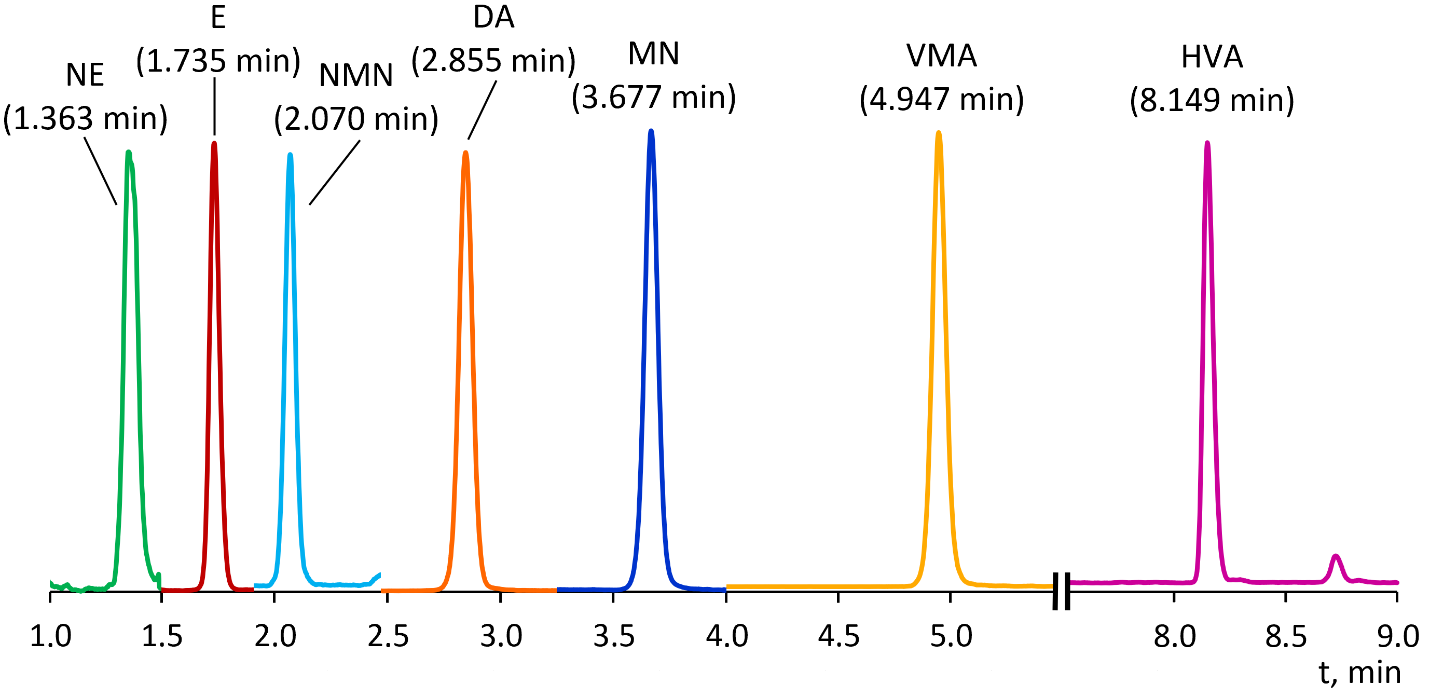


**Figure S3.** Multiple reaction monitoring (MRM) chromatograms of a pre-spiked urine sample after molecularly imprinted solid-phase extraction. Spiked sample concentrations: 5.1 μg/L norepinephrine; 3.7 μg/L epinephrine; 5.5 μg/L normetanephrine; 30.6 μg/L dopamine; 3.9 μg/L metanephrine; 0.48 mg/L vanillylmandelic acid; 0.44 mg/L homovanillic acid. Signal intensities not to scale
